# Supplementary material for: Theory of Large Intrinsic Spin Hall Effect in Iridate Semimetals
Source: Sci Rep. 2018 May 23;8:8052. doi: 10.1038/s41598-018-26355-y (PMC5966394; doi:10.1038/s41598-018-26355-y)
Supplement: Supplementary file 1 — Supplementary Information [file 41598_2018_26355_MOESM1_ESM.pdf]

Theory of Large Intrinsic Spin Hall Effect in Iridate Semimetals

Adarsh S. Patri[1], Kyusung Hwang[1,2], Hyun-Woo Lee[1,3], and Yong Baek Kim[1,*]

[1]Department of Physics and Centre for Quantum Materials, University of Toronto, Toronto, Ontario M5S 1A7, Canada
[2]Department of Physics, The Ohio State University, Columbus, OH 43210, USA
[3]PCTP and Department of Physics, Pohang University of Science and Technology, Pohang 37673, Korea
[*]ybkim@physics.utoronto.ca

Supplementary Information

Supplementary A : Hamiltonian matrix

The explicit form of the tight binding model[1,2] is given by the 8×8 Hamiltonian matrix:

$$H_{\mathbf{k}} = \left(\varepsilon^{po}_{r,\mathbf{k}}\sigma_y + \varepsilon^{po}_{i,\mathbf{k}}\sigma_x\right)\nu_z\tau_y + \left(\varepsilon^{zo}_{r,\mathbf{k}}\sigma_y + \varepsilon^{zo}_{i,\mathbf{k}}\sigma_x\right)\nu_y\tau_z + \left(\varepsilon^{do}_{r,\mathbf{k}}\sigma_y + \varepsilon^{do}_{i,\mathbf{k}}\sigma_x\right)\nu_x\tau_y + \varepsilon^{d}_{r,\mathbf{k}}\nu_x\tau_x + \varepsilon^{d}_{i,\mathbf{k}}\nu_y\tau_y + \varepsilon^{p}_{r,\mathbf{k}}\tau_x + \varepsilon^{p}_{i,\mathbf{k}}\sigma_z\tau_y + \varepsilon^{z}_{\mathbf{k}}\nu_x + \lambda_{\mathbf{k}}. \tag{S1}$$

Here the Pauli matrices σ, τ, ν act on the spin (↑,↓) and sublattice (1,2,3,4) degrees of freedom in the following way.

$$\sigma_x : \uparrow \leftrightarrow \downarrow; \qquad \nu_x: \ 1 \leftrightarrow 3,\ 2 \leftrightarrow 4; \qquad \tau_x: \ 1 \leftrightarrow 2,\ 3 \leftrightarrow 4. \tag{S2}$$

The coefficients of $H_{\mathbf{k}}$ are listed in Table S1. To help understanding of the model Hamiltonian, we visualize electron hopping channels in Fig. S1 with a detailed explanation about the model in the caption.

	Spin-indep.	Spin-dep.
NN	$\lambda_{\mathbf{k}} = t_{xy}\cos k_x \cos k_y$<br>$\varepsilon^{p}_{r,\mathbf{k}} = 2t_p(\cos k_x + \cos k_y)$<br>$\varepsilon^{z}_{\mathbf{k}} = 2t_z \cos k_z$	$\varepsilon^{p}_{i,\mathbf{k}} = -t'_p(\cos k_x + \cos k_y)$<br>$\varepsilon^{zo}_{r,\mathbf{k}} = t^o_z \cos k_z$<br>$\varepsilon^{zo}_{i,\mathbf{k}} = -t^o_z \cos k_z$<br>$\varepsilon^{po}_{r,\mathbf{k}} = t^o_{1p}\cos k_x + t^o_{2p}\cos k_y$<br>$\varepsilon^{po}_{i,\mathbf{k}} = -t^o_{2p}\cos k_x - t^o_{1p}\cos k_y$
NNN	$\varepsilon^{d}_{r,\mathbf{k}} = t_d(\cos k_x + \cos k_y)\cos k_z$<br>$\varepsilon^{d}_{i,\mathbf{k}} = t'_d(\sin k_x + \sin k_y)\sin k_z$	$\varepsilon^{do}_{r,\mathbf{k}} = t^o_d \sin k_y \sin k_z$<br>$\varepsilon^{do}_{i,\mathbf{k}} = t^o_d \sin k_x \sin k_z$

Table S1. Coefficients of $H_{\mathbf{k}}$ organized into first-nearest neighbour (NN) and second-nearest neighbour (NNN), as well as spin-independent and spin-dependent, hoppings. Here $k_x \equiv \mathbf{k}\cdot\mathbf{x}$, $k_y \equiv \mathbf{k}\cdot\mathbf{y}$, $k_z \equiv \mathbf{k}\cdot\mathbf{z}$, and $\{t_p, t_z, t_{xy}, t_d, t'_d, t'_p, t^o_{1p}, t^o_{2p}, t^o_z, t^o_d\}$ are hopping parameters. The values of the hopping parameters used in our calculations are listed in Table S2.

t_p	t'_p	t_{xy}	t_z	t^0_z	t_d	t'_d	t^0_{1p}	t^0_{2p}	t^0_d
-0.6	-0.15	-0.3	-0.6	0.13	-0.3	0.03	0.1	0.3	0.06

Table S2. Hopping parameters of the tight-binding model in the unit of eV[1,2].

Supplementary B : Representations of symmetry operators

Here we provide representations of symmetry operations on electron operators.

$$\begin{aligned}
G_n: &\quad \psi_{\mathbf{k}} \rightarrow \frac{i}{\sqrt{2}} e^{i\frac{k_a - k_b + k_c}{2}} (\sigma_x - \sigma_y)\nu_x\tau_x \psi_{(k_a,-k_b,k_c)},\\
G_b: &\quad \psi_{\mathbf{k}} \rightarrow -\frac{i}{\sqrt{2}} e^{i\frac{-k_a + k_b}{2}} (\sigma_x + \sigma_y)\tau_x \psi_{(-k_a,k_b,k_c)},\\
S_a: &\quad \psi_{\mathbf{k}} \rightarrow -\frac{i}{\sqrt{2}} e^{i\frac{k_a - k_b}{2}} (\sigma_x + \sigma_y)\tau_x \psi_{(k_a,-k_b,-k_c)},\\
\bar{I}: &\quad \psi_{\mathbf{k}} \rightarrow \psi_{(-k_a,-k_b,-k_c)},\\
T: &\quad \psi_{\mathbf{k}} \rightarrow -i\sigma_y K \psi_{(-k_a,-k_b,-k_c)},\\
m: &\quad \psi_{\mathbf{k}} \rightarrow -ie^{-ik_c/2}\sigma_z\nu_x \psi_{(k_a,k_b,-k_c)},
\end{aligned} \tag{S3}$$

where σ, μ, τ represent the Pauli matrices acting on spin and sublattice degrees of freedom, and K means complex conjugation. In these representations, phase factors can vary depending on the gauge choice for the electron operators $\psi_{\mathbf{k}}$. Despite such gauge dependence, phase factors contain important physical information such as the commutation relations in Eq. S4.

Supplementary C : Symmetry-protected nodal line

We briefly review the mechanism of the symmetry-protected nodal line[3] for self-containedness in our discussions. The nodal line band crossing (or nodal ring) occurs by the interplay of the three nonsymmorphic symmetries, n-glide plane (G_n), b-glide plane (G_b), and a-screw axis (S_a), and its symmetry protection can be understood by investigating the little group of the Hamiltonian matrix $H_{\mathbf{k}}$.

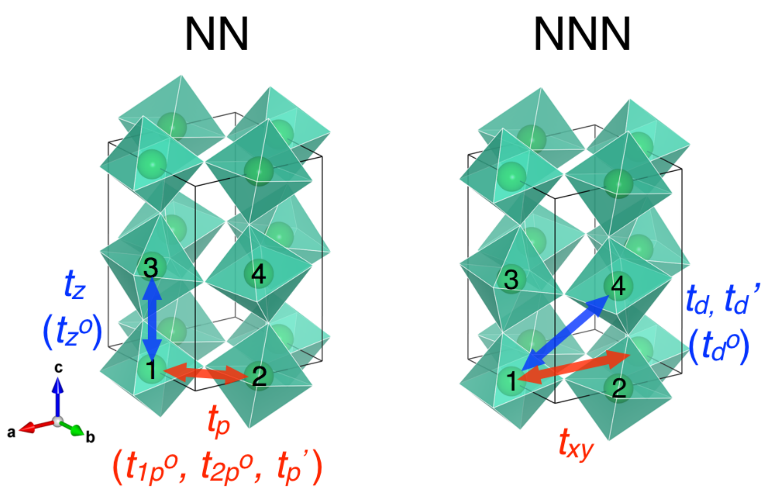

Figure S1. Nearest-neighbor (NN) and next-nearest-neighbor (NNN) hopping channels for the $j_{\text{eff}} = 1/2$ Ir electrons. Ten hopping channels corresponding to the parameters $\{t_p, t_z, t_{xy}, t_d, t_d', t_p', t_{1p}^o, t_{2p}^o, t_z^o, t_d^o\}$ are classified based on whether they occur along an in-plane (red) or out-of-plane (blue) direction, and also whether they are spin-independent or spin-dependent (spin-dependent hopping channels are denoted within the parentheses). The hopping model is an effective description of more microscopic, direct hopping between iridium sites as well as indirect hopping through intermediate oxygen sites. The spin-dependent hopping channels $\{t_p', t_{1p}^o, t_{2p}^o, t_z^o, t_d^o\}$ of the $j_{\text{eff}} = 1/2$ model are consequences of the rotation and/or tilting of the oxygen octahedra in the orthorhombic perovskite structure of $SrIrO_3$. Particularly, t_d^o plays an important role for large spin Hall effect in this system by controlling the size of the nodal ring as well as the spacing between the nearly degenerate bands as already explained in the main text.

First, we specify the little group for the $k_b = \pi$ plane where the nodal line appears. Under a space group symmetry operation, Bloch states (momentum eigenstates) generally move from a $\mathbf{k}$ point to another in the Brillouin zone unless the operation is a pure translation. Nevertheless, at high symmetry points of the Brillouin zone, the momentum of Bloch states can be invariant under certain symmetry operations. Such symmetry operations define the little group of $H_{\mathbf{k}}$ at a given high symmetry point. In the case of the *Pbnm* space group, the entire $k_b = \pi$ plane is invariant under G_n. Moreover, high symmetry points on that plane such as the U point and RS and SX lines have further little group elements (G_b and/or S_a) as summarized in Table S3. One can check this from the transformation rules of electron operators in Eq. S3. Moreover, we can show that $G_n^2 = -T_{\mathbf{a}+\mathbf{c}}$, $G_b^2 = -T_{\mathbf{b}}$, $S_a^2 = -T_{\mathbf{a}}$ (where $T_{\mathbf{r}}$ represents a translation by a lattice vector $\mathbf{r}$, and the minus sign in each case arises due to a

	$\mathbf{k}$	G_n	G_b	S_a
$k_b = \pi$ plane	(k_a, π, k_c)	✓		
U point	$(0, \pi, \pi)$	✓	✓	✓
RS line	(π, π, k_c)	✓	✓	
SX line	$(k_a, \pi, 0)$	✓		✓

Table S3. Elements of the little group of $H_{\mathbf{k}}$ at various high symmetry locations in the Brillouin zone. In each case, little group elements are denoted with checkmarks.

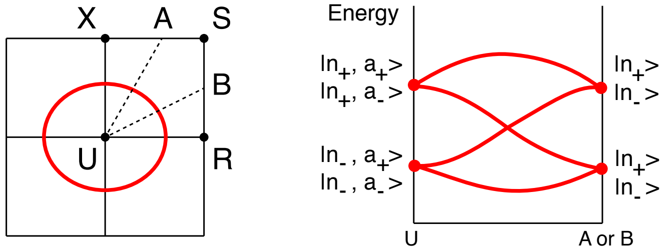

Figure S2. Schematic illustration of the mechanism on the symmetry-protected nodal line. In the left figure, the red circle represents the nodal ring on the $k_b = \pi$ plane. The right figure describes how the G_n-eigenvalue structure of the energy bands changes along paths connecting the U point with the RS or SX line (dashed lines in the left figure).

2π-rotation of $j_{\text{eff}} = 1/2$ spin). This tells us the eigenvalues of $\{G_n, G_b, S_a\}$: $n_\pm \equiv \pm ie^{i(k_a+k_c)/2}$ for G_n, $b_\pm \equiv \pm ie^{ik_b/2}$ for G_b, and $a_\pm \equiv \pm ie^{ik_a/2}$ for S_a. Here it is important to notice that $G_n(= n_\pm)$ serves as a good quantum number to specify Bloch states over the whole $k_b = \pi$ plane.

Now we consider the commutation relations of the little group elements listed in Table S3. By using Eq. S3, we can find the commutation relations for the U point and RS and SX lines as follows.

$$\begin{aligned} &(\text{U}) \quad [G_n, G_b] = [G_n, S_a] = \{G_b, S_a\} = 0, \\ &(\text{RS}) \quad \{G_n, G_b\} = 0, \\ &(\text{SX}) \quad \{G_n, S_a\} = 0. \end{aligned} \tag{S4}$$

The anti-commutation relations impose constraints on electron band structure: energy levels at the high symmetry point and lines must be at least fourfold-degenerate due to the anti-commutativeness and the Kramers degeneracy. The minimal fourfold degeneracy is actually observed in the band structure shown in Fig. 2 (two fourfold-degenerate bands at the U point and along the RS and XS lines) in the main text.

More importantly, the anti-commutation relations determine the G_n-eigenvalue structure within each fourfold-degenerate energy level. As illustrated in Fig. S2, the two levels at the U point are characterized by the different eigenvalues: n_+ for the upper and n_- for the lower. The upper level consists of four states $\{|n_+, a_+\rangle, |n_+, a_-\rangle, \Theta|n_+, a_+\rangle, \Theta|n_+, a_-\rangle\}$ which are simultaneous eigenstates of G_n and S_a. Here Θ is the product of time-reversal and spatial-inversion, and it satisfies $[\Theta, G_n] = 0$. These states form a four dimensional representation with the little group structure stated in Eq. S4. In this representation, both a_+ and a_- eigenstates are required by the relation $\{G_b, S_a\} = 0$; under the G_b operation, a_+ state is mapped into a_- state and vice versa ($|a_+\rangle \overset{G_b}{\leftrightarrow} |a_-\rangle$). Similarly, the lower level is formed by four states $\{|n_-, a_+\rangle, |n_-, a_-\rangle, \Theta|n_-, a_+\rangle, \Theta|n_-, a_-\rangle\}$ which realize another four dimensional representation of the little group. However, there is no symmetry requirement that n_+ and n_- eigenstates must coexist in each of the two energy levels at the U point.

Along the SX line, each level comprises four states $\{|n_+\rangle, |n_-\rangle, \Theta|n_+\rangle, \Theta|n_-\rangle\}$ due to the relation $\{G_n, S_a\} = 0$ ($\Rightarrow |n_+\rangle \overset{S_a}{\leftrightarrow} |n_-\rangle$). Here we stress that both n_+ and n_- eigenstate appear in each level, in contrast to the case at the U point. This means that there must be G_n-partner exchange between the upper and lower levels and thereby band crossing between the two bands involved in the partner exchange, along any path from the U point to the SX line (see Fig. S2). Similar argument works for any path connecting the U point with the RS line, in which case the relation $\{G_n, G_b\} = 0$ leads to the coexistence of n_+ and n_- states in each of two energy levels.

Supplementary D : Signature of nonsymmorphic symmetries in bulk SHC

Our results show that the system exhibits large spin Hall response when the spin current is induced along the z axis (σ^y_{zx} and σ^y_{zy} in Fig. 2 in the main text). Keeping the spin current direction along the z axis, we change the applied electric field direction to investigate the field direction dependence of spin Hall conductivity. Specifically, we consider the following configuration.

$$\begin{aligned} \nu &\parallel \hat{x}\cos\theta + \hat{y}\sin\theta, \\ \mu &\parallel \hat{z}, \\ \rho &\parallel \hat{x}\sin\theta - \hat{y}\cos\theta. \end{aligned} \tag{S5}$$

Here, the field direction (ν) is changed within the xy plane by the angle θ from the x axis with keeping the three directions $\{\rho, \mu, \nu\}$ orthogonal. In this setting, by the nonsymmorphic symmetries, the spin Hall conductivity is simply a combination of σ^y_{zx} and σ^y_{zy}:

$$\sigma^\rho_{\mu\nu} = -\sigma^y_{zx} - \sigma^y_{zy}\sin 2\theta. \tag{S6}$$

As described in Fig. S3, the spin Hall conductivity drastically changes around the zero Fermi energy as the field direction varies.

The largest magnitude of spin Hall conductivity occurs when $\theta = 45^\circ$ and $\theta = 135^\circ$ which correspond to the $[110]_c$ and $[1\bar{1}0]_c$ pseudo-cubic axes for the field direction, respectively (those two axes are identical to the $[100]_o$ and $[0\bar{1}0]_o$ orthorhombic axes). For the experiments on the bulk system, this suggests that the largest spin Hall response is expected for the electric field along the $[100]_o$ or $[0\bar{1}0]_o$ axis. On the other hand, the characteristic sinusoidal angular dependence (inset of Fig. S3) can be used to experimentally probe the presence of the nonsymmorphic symmetries in the system. The sinusoidal behaviour described in Eq. S6 is dictated by the *Pbnm* nonsymmorphic symmetries regardless of the details of the Hamiltonian.

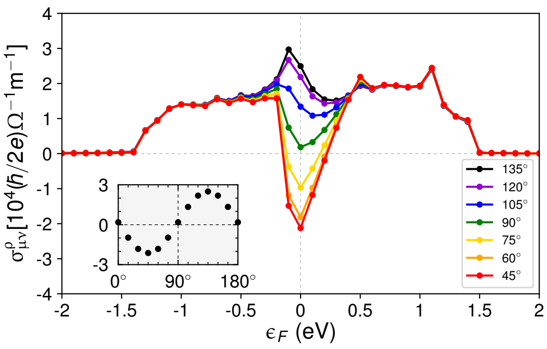

Figure S3. Field direction dependence of spin Hall conductivity. Inset: angular dependence of spin Hall conductivity at $\varepsilon_F = 0$.

Supplementary E : Influence of perturbations on bulk band structure

In this section, we depict the impact of the symmetry breaking perturbations (h_{gap}, h_{xx}, and h_{xz}) on the band structure (along certain high symmetry directions). In particular, one can see in Fig. S4 that the introduction of h_{gap} gaps out the nodal ring.

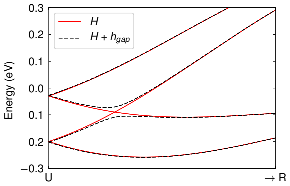

(a) UR Direction

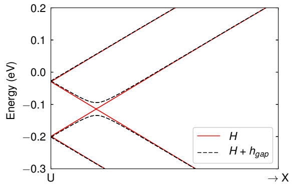

(b) UX Direction

Figure S4. Gapped nodal ring band structure due to h_{gap} with $t_{gap} = 0.01$eV

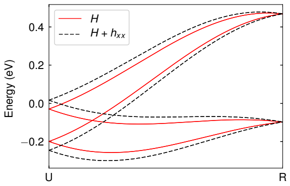

(a) UR Direction

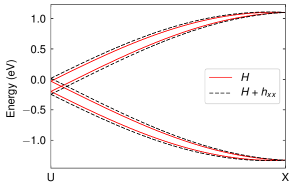

(b) UX Direction

Figure S5. Perturbed band structure due to h_{xx} with $t_{xx} = 0.05$eV

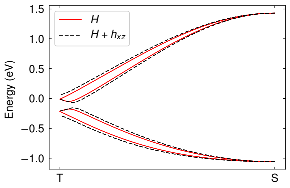

(a) TS Direction

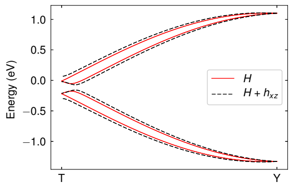

(b) TY Direction

Figure S6. Perturbed band structure due to h_{xz} with $t_{xz} = 0.05$eV

Supplementary F : Thin film electron distribution over layers of Ir sites

To understand the difference between the thin film and bulk systems (and hence the origin of the large thin film spin Hall conductivity), we studied the distribution of the electron density over the layers of the Ir sites. Below, in Fig. S7 we present the electron density as a function of layer number. The lack of uniformity (in the electron density) over the layers is a manifestation of lower lattice symmetry in the thin film (in contrast to the bulk system).

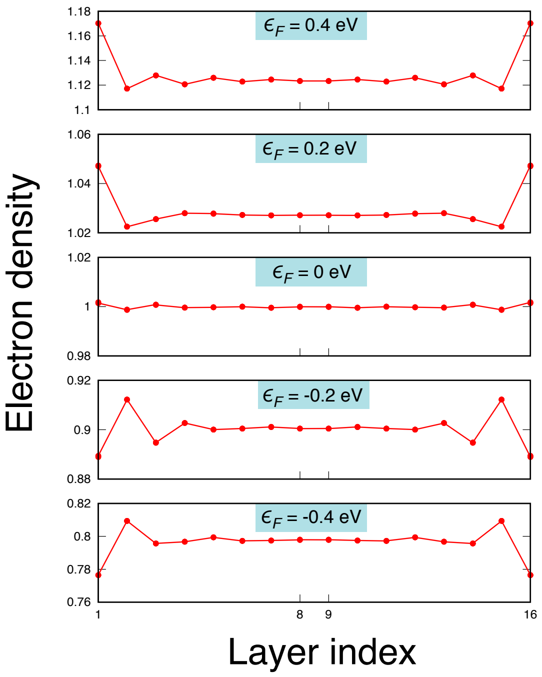

Figure S7. Layer dependence of electron density in the thin film system. The layer index represents the 16 Ir-layers of the film system ($1 \sim 16$: top to bottom layer). The symmetric pattern of the electron density about the central layers (8,9) is due to the inversion symmetry remaining in the film system.

Supplementary G : Influence of magnetic perturbations on bulk spin Hall effect

In the main text, we considered the influence of non-magnetic perturbations to determine the stability of the spin Hall conductivity. However as discussed in Sec. 6 in the main text, a ferromagnetic permalloy, Py, (situated nearby to the $SrIrO_3$ thin film) has an exchange field that could potentially leak into the $SrIrO_3$. The Py plays an important role as it is used in the actual measurement of the spin Hall effect: the spin current which flows into the Py (from the spin Hall effect in $SrIrO_3$) exerts a torque on the magnetic moment causing it to precess. The precession leads to a voltage being generated (via the anisotropic magnetoresistance) in the Py which is measured. Our goal here is to incorporate the effects of the Py's exchange field into our calculations. In order to get an estimate for the strength of the ferromagnetic exchange field, one notes that the exchange stiffness constant in permalloy to be $\mathscr{A}_{exchange} = 1.3 \times 10^{-11} J/m$[4] and the lattice constant to be $a = 3.55$ Å[5]. Hence, an estimate of the exchange constant is $J_{exchange} \approx \mathscr{A} a = 0.0288 eV$. This corresponds to an exchange field of approximately $B_{ex} \approx 490T$. It is important to note that since the actual exchange field is localized at the interface, as a simple approximation we simulate its impact as a constant Zeeman field that acts uniformly on the bulk. Moreover, we take the strength of this Zeeman field to be much weaker than the actual exchange field to incorporate the fact that the exchange field is localized at the interface and gets weaker for distances further away from the interface: $B_{Zeeman} = 0.01$ eV $\leftrightarrow \approx$ 170T. This Zeeman magnetic field perturbation also serves as a means to determine the robustness of the spin Hall effect in $SrIrO_3$ to large, stray external magnetic fields. Since the direction of the exchange field from the permalloy can be arbitrary, we examine the effects of the Zeeman field pointing along each one of the orthorhombic axes: $\{\vec{a}, \vec{b}, \vec{c}\}$. As has been previously described[2], the nodal ring undergoes different experiences depending on the direction of the external magnetic field. If the magnetic field is along the $\vec{c}$ axis, the chiral symmetry is broken and the nodal ring is gapped out (in addition to the two-fold degeneracy being broken due to broken time-reversal symmetry). If the magnetic field is along the $\vec{b}$ axis, the doubly degenerate nodal ring splits into two non-degenerate nodal rings shifted along the $\vec{c}$ axis. Finally, if the magnetic field is along the $\vec{a}$ axis, the nodal ring is replaced

by 3D Dirac nodes (which are not symmetry protected). Below we present the impact of the nodal ring calculation on the σ_{zx}^{y} and σ_{xz}^{x} components in Fig. S8(a), (c) respectively.

The spin Hall conductivity is stable to the introduction of magnetic fields along the $\vec{a}$ and $\vec{b}$ axes. A magnetic field along the $\vec{c}$ axis does not change the spin Hall conductivity substantially for all Fermi levels except near the zero energy level. In particular, at the zero energy level, σ_{zx}^{y} increases by about an order of magnitude, while σ_{xz}^{x} increases by almost 2.5 times. To discern the cause for this, we once again examine the momentum-resolved SHC at the zero Fermi level in Fig. S8(b), (d). There appear to be new features that develop throughout the Brillouin zone (rings around the U and T points, as well as a circular loop about the zone centre) that lead this large change in the spin Hall conductivity. Although this increase is very promising, one should recall again that in a realistic system the exchange field is localized near the boundary. Nevertheless, one can still appreciate that the spin Hall conductivity is stable to such large magnetic fields for most of the Fermi levels (except near the zero energy level). Near the zero energy level, these results suggest that the exchange field can indeed induce a large change in the spin Hall conductivity.

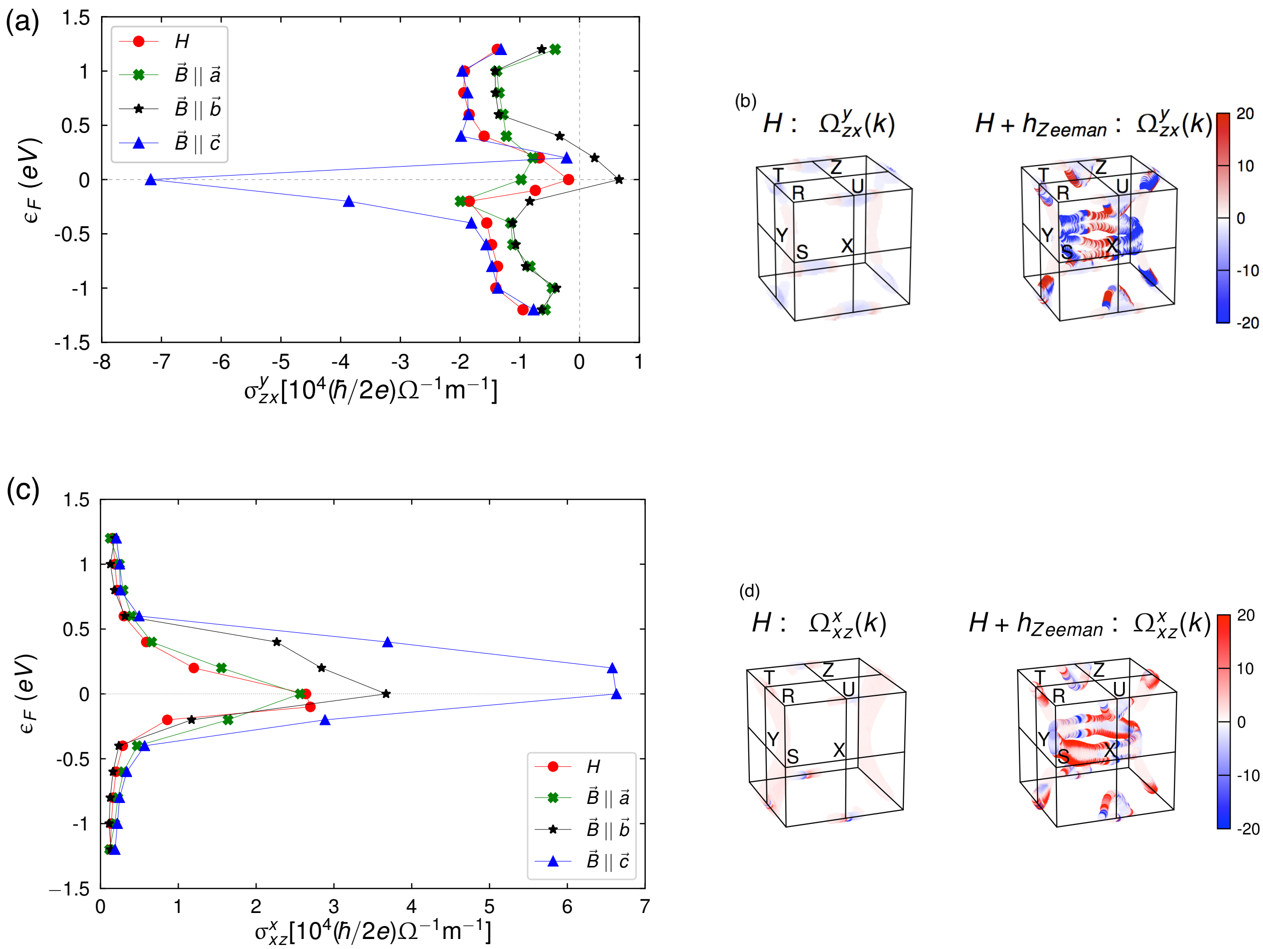

Figure S8. (a),(c) SHC of original bulk model (H) compared to bulk model augmented by magnetic (Zeeman) perturbations. (b),(d) Momentum-resolved SHC at the zero Fermi level for σ_{zx}^{y} and σ_{xz}^{x}, respectively, for original bulk model (H) compared to bulk model augmented by magnetic (Zeeman) perturbations along the $\vec{c}$ direction.

References

1. Carter, J.-M., Shankar, V. V., Zeb, M. A. & Kee, H.-Y. Semimetal and Topological Insulator in Perovskite Iridates. *Phys. Rev. B* **85**, 115105 (2012).
2. Chen, Y., Lu, Y.-M. & Kee, H.-Y. Topological crystalline metal in orthorhombic perovskite iridates. *Nat. Commun.* **6**, 6593 (2015).

3. Chen, Y., Kim, H.-S. & Kee, H.-Y. Topological crystalline semimetals in nonsymmorphic lattices. *Phys. Rev. B* **93**, 155140 (2016).

4. Hertel, R. Thickness dependence of magnetization structures in thin Permalloy rectangles. *Zeitschrift für Met.* **93**, 957–962 (2002).

5. Yu, P., Jin, X. F., Kudrnovský, J., Wang, D. S. & Bruno, P. Curie temperatures of fcc and bcc nickel and permalloy: Supercell and Green's function methods. *Phys. Rev. B* **77**, 054431 (2008).
